# Effects of All-Trans Retinoic Acid on Ovarian Development, Lipid Metabolism, Nutritional Quality, and Gut Microbiota of Female Chinese Mitten Crab During Fattening Period

**DOI:** 10.3390/ijms27115148

**Published:** 2026-06-05

**Authors:** Peng Huang, Jiancao Gao, Jinliang Du, Haojun Zhu, Liping Cao, Jun Gao, Jiayi Li, Yao Zheng, Gangchun Xu, Shunlong Meng

**Affiliations:** 1 Wuxi Fisheries College, Nanjing Agricultural University, Wuxi 214081, China; 2024213005@stu.njau.edu.cn (P.H.); gaojiancao@ffrc.cn (J.G.); dujl@ffrc.cn (J.D.); caolp@ffrc.cn (L.C.); zhengy@ffrc.cn (Y.Z.); 2 Freshwater Fisheries Research Center, Chinese Academy of Fishery Sciences, Wuxi 214081, China; zhuhaojun@ffrc.cn (H.Z.); gaojun@ffrc.cn (J.G.); lijiayi@ffrc.cn (J.L.)

**Keywords:** retinoic acid, *Eriocheir sinensis*, multiomics, oocyte development, in vivo injection, dietary supplementation, lipid metabolism

## Abstract

All-trans retinoic acid (atRA) is known to regulate lipid metabolism, adipocyte differentiation, and the immune system in mammals and other aquatic species. However, studies on atRA in crustaceans, especially in *Eriocheir sinensis*, are still scarce. The present study aimed to investigate the regulatory effects of dietary or injected atRA on female crabs during the fattening period. In the dietary regulation experiment, 270 female crabs were fed diets containing different doses of atRA (0, 150, 300, 600, 1200, and 2400 mg/kg) for a total of 49 days. In the in vivo injection experiment, 90 females were divided into an experimental group (injected with a 0.3 μg/g dose of atRA) and a control group (injected with the same amount of DMSO solvent). Injections were given weekly throughout the 35-day experimental period. Results: Both dietary atRA and atRA injection promoted ovarian development, as evidenced by increased GSI, elevated serum Vg levels, decreased GIH, and upregulated expression of *vg*, *vgr*, and *rxr* genes. In terms of mechanism, dietary atRA promoted ovarian development via the upregulation of pyrimidine nucleotides and dehydroepiandrosterone, which supplied nucleic acid precursors and hormonal support. Furthermore, RXR was identified as a potential key target of atRA in inducing ovarian development, as molecular docking revealed that atRA could spontaneously bind to RXR. Moreover, following atRA injection, the expression of *rxr*, along with key genes involved in ovarian development, lipid synthesis, and lipid transport, was significantly upregulated. In addition, the atRA diet created a favorable microenvironment for ovarian development by reducing pro-inflammatory lipid levels in the ovary. Transcriptomic and metabolomic analyses revealed that atRA modulates energy and lipid metabolism by activating the AMPK pathway. In terms of the bacterial community structure, the atRA diet significantly decreased *Fusobacterium* abundance and enriched *Parabacteroides* as the signature beneficial bacterium. In terms of nutritional quality, the atRA diet markedly reduced saturated and trans-fatty acids while increasing monounsaturated fatty acids and various key essential amino acids. Conclusions: This study revealed that atRA plays a key role in promoting ovarian development, improving nutritional quality, and modulating the structure of the microbiota, thereby providing theoretical support for healthy aquaculture of female crabs during the fattening period.

## 1. Introduction

The Chinese mitten crab, *Eriocheir sinensis*, is widely distributed worldwide, with significant production in China. In China, the cultivation of *E. sinensis* has a wide coverage, forming production areas mainly in the middle and lower reaches of the Yangtze River and the Liao River Basin. Except for a few regions such as Beijing and Hainan, *E. sinensis* cultivation is carried out in all provinces across the country. In recent years, the annual total output of *E. sinensis* across the country has remained stable at around 8 million tons, providing important support for the stable production and supply of fishery products. As is well known, *E. sinensis* is a seasonal consumer product, mainly sold in its fresh state. Concentrated market releases, from October to November each year in China, lead to short supply cycles and low production benefits for commercial crabs. With the expansion of the production volume of *E. sinensis*, the drawback of a large number of concentrated market releases has become increasingly prominent. Early or delayed development of their gonads will help extend the shelf life and weaken the negative impact of concentrated market releases on the *E. sinensis* industry. After completing the reproductive molting, crabs enter the rapid development stage of their gonads. This stage corresponds to the fattening process in production. Therefore, conducting research on the regulation of gonadal development during fattening is of great significance.

It is well known that the demand for nutrients (protein and lipids) increases significantly to facilitate the rapid development of gonadal tissue during the fattening period. Compared to male crabs, female crabs have a higher gonadosomatic index (GSI) and a more unique nutritional flavor, making them highly favored by breeders and consumers. In females, the rapid growth of oocytes and yolk accumulation heavily relies on vitellogenin (Vg) produced by the hepatopancreas [1]. The hepatopancreas serves as a vital organ responsible for nutrient absorption and storage, as well as supplying necessary substances and energy in *E. sinensis* [2,3]. Furthermore, the hepatopancreas exhibits comprehensive and stable nutritional profiles throughout the processes of reproductive molting and gonadal development. Thus, the nutritional composition of the hepatopancreas is the key factor determining the quality of *E. sinensis*.

Retinoic acid, a metabolite of vitamin A, is the most active among natural vitamin A compounds. Several isoforms exhibit different functions in specific organs, including all-trans retinoic acid (atRA), 9-cis retinoic acid (9-cis RA), and 13-cis retinoic acid (13-cis RA) [4]. The known function of vitamin A acid is mediated through specific nuclear receptors, the retinoic acid receptor (RAR) and retinoid X receptor (RXR) in target cells. Notably, atRA acts as the natural ligand of both RAR and RXR [5,6,7]. Compared to cis-retinoic acid, atRA has the highest content in natural tissues and is also the main metabolic product of vitamin A. Due to its easy availability and relatively low cost, atRA has become the primary type of retinoic acid studied as a feed additive. Numerous studies have confirmed that atRA plays a crucial role in biological processes such as lipid metabolism, adipocyte differentiation, and the immune system, with validation in mammals (mice, rabbits, and cattle) and the aquatic crustacean giant freshwater prawn [8,9,10,11,12]. In mice, the gut microbiota plays a crucial role in retinol metabolism by regulating immune function, lipid metabolism, and other nutrient metabolisms [13]. Notably, exogenous atRA treatment can facilitate gonadal development during the embryonic development of *Macrobrachium rosenbergii* [14].

Our previous study demonstrated that long-term low-temperature stimulation during the fattening period of female crabs can significantly promote oocyte development, and this mechanism is presumably associated with the activation of the key gene *rxr* [15]. Besides being a potential natural agonist of RXR, in crustaceans, atRA can regulate lipid metabolism and ovarian development through the peroxisome proliferator-activated receptor (PPAR) and ecdysone receptor (EcR) pathways, respectively [16,17]. Based on these established findings, we propose the hypothesis: in *E. sinensis*, atRA may participate in regulation by binding to RXR, and it may also regulate lipid metabolism through specific signaling pathways, ultimately promoting ovarian development. Beyond the well-documented gene-level regulatory pattern, we innovatively hypothesize and partially verify that atRA can provide critical biosynthetic substrates (including nucleotides and sex hormone precursors) for ovarian development, supplementing the material metabolic regulatory mechanism of atRA. More importantly, this study is the first to demonstrate that dietary atRA effectively improves the nutritional quality and remodels the intestinal microbiota structure of *E. sinensis*, which represents an unreported novel finding in related research fields. Overall, the present study investigated the all-around impacts of dietary or injected atRA on *E. sinensis*. The results of this study help to fill the current research gap in the field of atRA-mediated nutritional regulation in *E. sinensis*. Meanwhile, it provides a theoretical reference and technical inspiration for the off-peak marketing of *E. sinensis*.

## 2. Results

### 2.1. The Effects of an atRA Diet on Oocyte Morphology, Hemolymph-Related Enzymes, and Hormones

**Figure 1:**
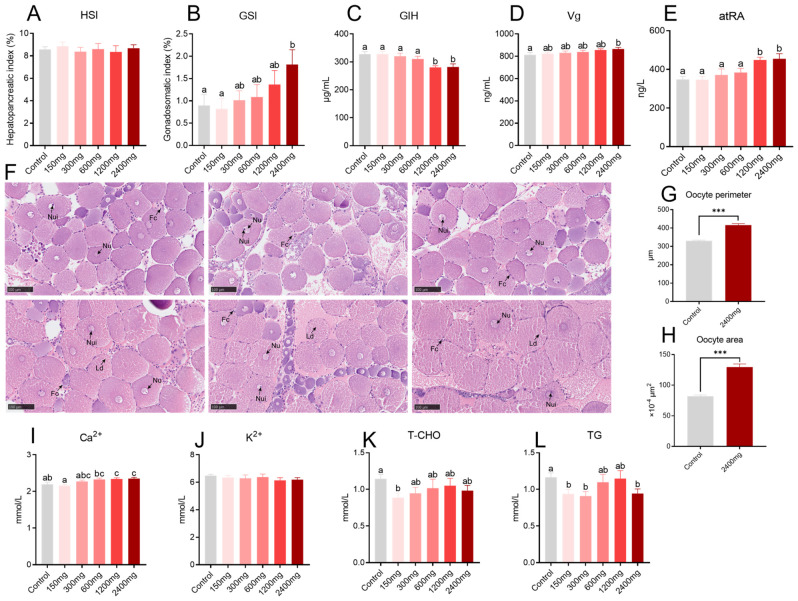
Effects of dietary atRA on the morphology of oocytes, hemolymph-related enzyme activities, and hormones in female crabs after reproductive molting (*n* = 15). (**A**) Hepatopancreas index (HSI). (**B**) Gonadosomatic index (GSI). (**C**) Hemolymph gonad-inhibiting hormone (GIH) content. (**D**) Hemolymph vitellogenin (Vg) content. (**E**) Hemolymph atRA content. (**F**) Hematoxylin-eosin (H&E) staining analysis of oocytes, with the first row representing the control group and the second row indicating the 2400 mg/kg atRA experimental group (scale bar: 100 μm). Nu, nucleus; Nui, nucleolus; Fc, follicular cell; Ld, lipid droplet. (**G**,**H**) Calculation of oocyte perimeter and area based on NDP.view2 (v2.7.52) software, respectively. (**I**–**L**) The content or concentration of Ca^2+^, K^2+^, TG, and T-CHO in the hemolymph, respectively. Different lowercase letters indicate significant differences between groups based on one-way ANOVA (*p* < 0.05), and “***” indicates extremely significant differences between two groups based on Student’s *t*-test (*p* < 0.001).

There was no significant difference in HSI between groups (Figure 1A), while GSI gradually increased with increasing atRA dosage, especially in the 2400 mg/kg dosage group, which was significantly higher than the control group (Figure 1B, *p* < 0.05). The content of GIH in the hemolymph significantly decreased in the 1200–2400 mg/kg group, while the content of Vg and atRA significantly increased in the 2400 mg/kg group (Figure 1C–E, *p* < 0.05). H&E analysis results revealed that the oocyte granulometric size (including perimeter and area) in the highest dosage group increased significantly (Figure 1F–H, *p* < 0.001). Furthermore, compared to the control group, the highest dosage group had significantly increased Ca^2+^ content in the hemolymph, significantly decreased TG, and a slight decrease in T-CHO (Figure 1I–L).

### 2.2. The Effect of the atRA Diet on the Transcriptomic Profiles of Hepatopancreas and Ovary

**Figure 2:**
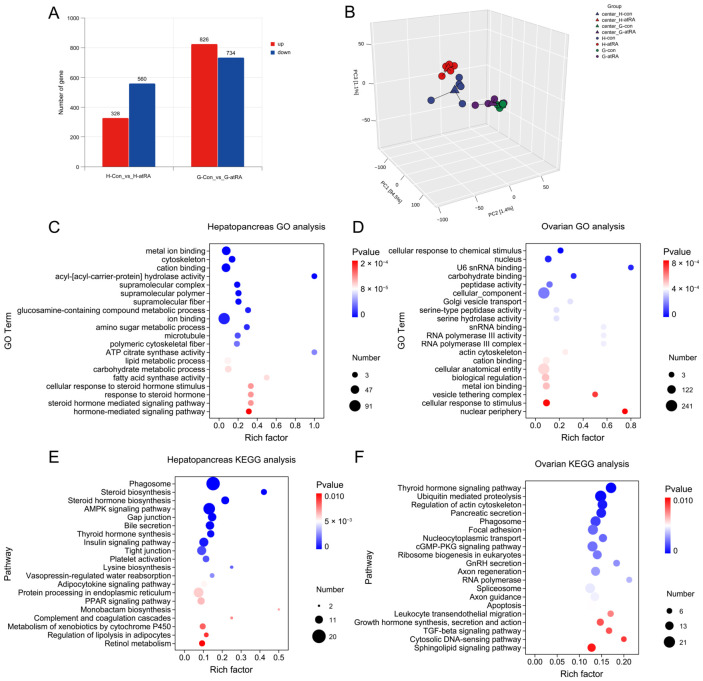
The effect of dietary atRA on the transcriptomic profiles of the hepatopancreas and ovary in female crabs (*n* = 5). (**A**) Volcano plots of differentially expressed genes (DEGs). (**B**) 3DPCA plots of samples. (**C**,**D**) GO enrichment analysis of DEGs in the hepatopancreas and ovary tissues, respectively. (**E**,**F**) KEGG enrichment analysis of DEGs in the hepatopancreas and ovary tissues, respectively. Differentially expressed genes (DEGs) were determined by FDR < 0.05. Raw *p*-values are displayed for reference. Same as below.

Compared to the control group, the atRA group exhibited a total of 888 (328 upregulated and 560 downregulated) and 1560 (826 upregulated and 734 downregulated) DEGs in the hepatopancreas and ovary tissues, respectively (Figure 2A,B). The PCA results indicated good intra-group reproducibility, satisfying the requirements for subsequent analysis (Figure 2B). GO enrichment analysis revealed that metal ion binding, the cytoskeleton, cation binding, etc., were significantly enriched in the hepatopancreas tissue, while cellular response to chemical stimulus, the nucleus, U6 snRNA binding, etc., were significantly enriched in the ovary tissue (Figure 2C,D). KEGG analysis showed that pathways related to lipid metabolism (steroid biosynthesis, PPAR, adipocytokine, regulation of lipolysis in adipocytes, and retinol metabolism), hormone synthesis and regulation (steroid hormone biosynthesis, thyroid hormone synthesis, and insulin), and the AMPK signaling pathway were significantly enriched in the hepatopancreas tissue (Figure 2E). In the ovary tissue, pathways related to cell signaling (thyroid hormone, cGMP-PKG, TGF-beta, sphingolipid, and the cytosolic DNA-sensing pathway), cytoskeleton and cell adhesion (regulation of actin cytoskeleton, focal adhesion, and leukocyte transendothelial migration), and hormone secretion and regulation (GnRH secretion; growth hormone (GH) synthesis, secretion, and action; and pancreatic secretion) were significantly enriched (Figure 2F).

**Figure 3:**
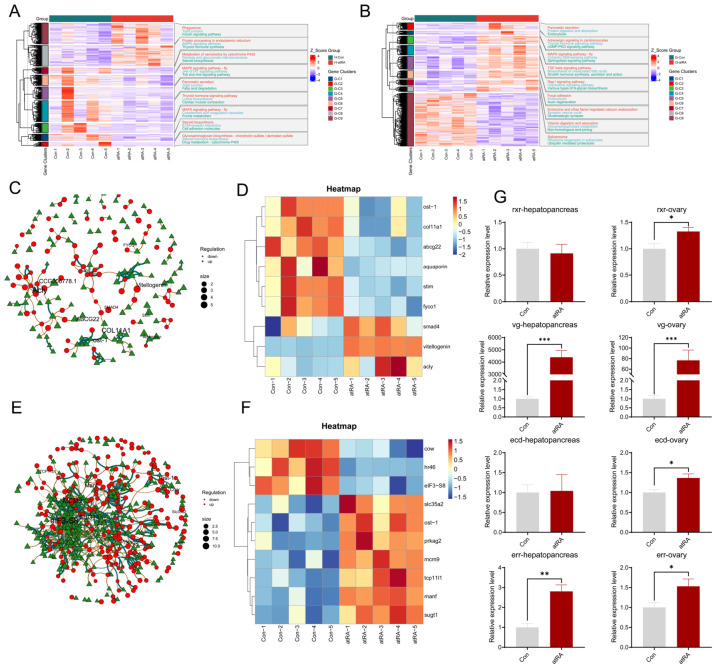
The effect of dietary atRA on the transcriptomic profiles of the hepatopancreas and ovary in female crabs (*n* = 5). (**A**,**B**) The heatmap and KEGG enrichment term plots generated based on the bidirectional cluster analysis between DEGs and samples in the hepatopancreas and ovary tissues, respectively. (**C**,**E**) Protein–protein interaction (PPI) network analysis in hepatopancreas and ovarian tissues, respectively. (**D**,**F**) Key DEGs screened by PPI in hepatopancreas and ovarian tissues, visualized through heatmaps. (**G**) The key DEGs related to ovarian development in transcriptomic results between hepatopancreas and ovarian tissues, with “*, **, ***” indicating significant differences between the two groups based on Student’s *t*-test analysis (*p* < 0.05 or *p* < 0.01 or *p* < 0.001).

Furthermore, the KEGG heatmap + enrichment bar graph revealed the expression patterns of DEGs or signaling pathways. Compared to the control group, gene clusters dominated by the phagosome and protein processing in the endoplasmic reticulum showed an upward trend in the atRA group, while gene clusters dominated by the metabolism of xenobiotics by cytochrome P450, MAPK, pancreatic secretion, thyroid hormone, and steroid biosynthesis tended to be downregulated (Figure 3A). Similarly, in ovarian tissue, most key pathways, including pancreatic secretion, adrenergic signaling in cardiomyocytes, MAPK, TGF-beta, Rap1, and focal adhesion, showed an upward trend (Figure 3B). Protein–protein interaction (PPI) network analysis revealed that the complexity of gene interactions in the hepatopancreatic tissue was significantly lower than that in the ovary. The heatmap results revealed the top 10 key DEGs, among which *acly*, vg, and *smad4* were significantly upregulated in the hepatopancreatic tissue, while *ost-1*, *col11a1*, *abcg22*, *aquaporin*, *stim*, and *fyco1* were significantly downregulated (Figure 3C,D). In ovarian tissue, *slc35a2*, *ost-1*, *prkag2*, *mcm9*, *tcp1111*, *manf*, and *sugt1* were significantly upregulated, while *cow*, *hr46*, and *elF3-S8* were significantly downregulated (Figure 3E,F). Furthermore, based on transcriptomic data, we analyzed key genes regulating ovarian development and found that *rxr* and *ecd* were only upregulated in ovarian tissue, while *vg* and *err* were significantly upregulated in different tissues, and *vg* expression in the hepatopancreatic tissue was over 4000 times higher than that in the control group (Figure 3G, *p* < 0.001).

### 2.3. The Effect of the atRA Diet on the Metabolomics of Hepatopancreas and Ovary

**Figure 4:**
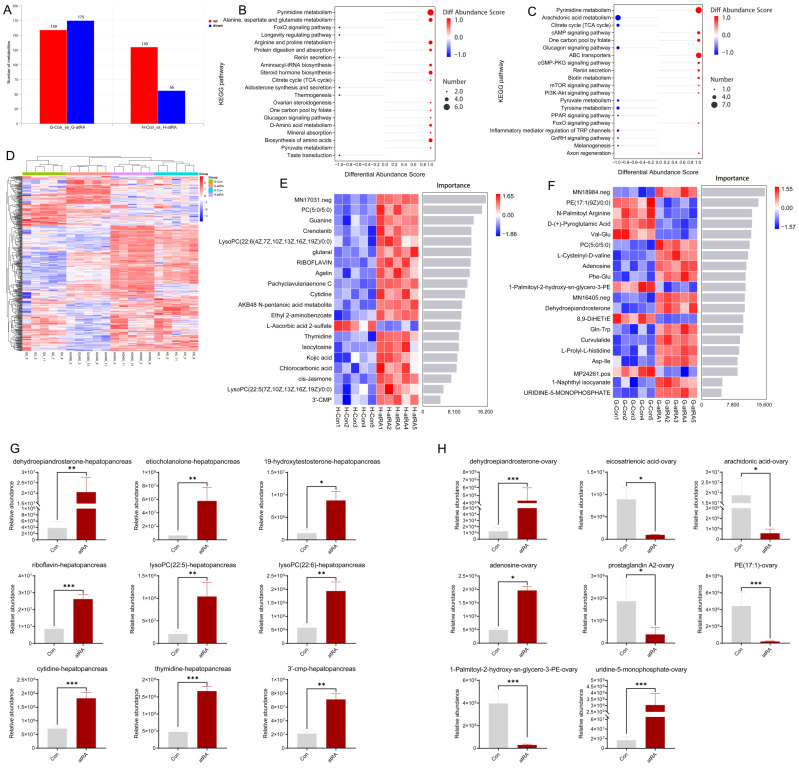
Effects of dietary atRA on the metabolomics of hepatopancreas and ovary in female crabs. (**A**) Differentially expressed metabolites (DEMs) in different tissues (*n* = 5). (**B**,**C**) KEGG pathway analysis of DEMs in hepatopancreas and ovary tissues, respectively. (**D**) Cluster heatmap analysis of DEMs. (**E**,**F**) Key DEMs screened based on random forest analysis in hepatopancreas and ovary tissues, respectively. (**G**,**H**) DEMs related to ovarian development screened in hepatopancreas and ovary tissues, respectively. “*, **, ***” indicate significant differences between two groups based on Student’s *t*-test analysis (*p* < 0.05 or *p* < 0.01 or *p* < 0.001).

As shown in Figure 4, there were 186 (130 upregulated and 56 downregulated) and 334 DEMs (159 upregulated and 175 downregulated) in the hepatopancreas and ovary, respectively (Figure 4A). The cluster heatmap of metabolites revealed good reproducibility between groups (Figure 4D). KEGG enrichment analysis of DEMs revealed that pathways involved in pyrimidine metabolism, amino acid biosynthesis and metabolism (including alanine, aspartate, and glutamate metabolism; arginine and proline metabolism; d-amino acid metabolism; and biosynthesis of amino acids), and hormone synthesis and secretion (steroid hormone biosynthesis, ovarian steroidogenesis) were significantly enriched and upregulated in the hepatopancreas tissue (Figure 4B). In the ovary, pyrimidine metabolism, cAMP, cGMP-PKG, mTOR, PI3K-Akt, FoxO, and other signaling pathways were significantly upregulated (Figure 4C). Furthermore, screening for key metabolites enriched in these pathways revealed that DEMs related to ovarian development and lipid metabolism (including dehydroepiandrosterone (DHEA), etiocholanolone, 19-hydroxytestosterone (19-OHT), lysoPC (22:5/6)) and pyrimidine nucleosides and nucleotide derivatives (cytidine, thymidine, cytidine 3′-monophosphate (3′-CMP)) were significantly upregulated in the hepatopancreas (Figure 4G). Among them, the DEMs of hepatopancreatic tissues showed a significant positive correlation with ovarian development (Figure S1). In the ovary tissue, besides significant upregulation of DHEA, adenosine, and uridine-5′-monophosphate (5′-UMP), other metabolites, including eicosatrienoic acid (ETA), arachidonic acid (ARA), prostaglandin A2 (PGA2), and PE (17:1), were significantly downregulated (Figure 4H).

### 2.4. The Effect of the atRA Diet on the Intestinal Microbial Community

**Figure 5:**
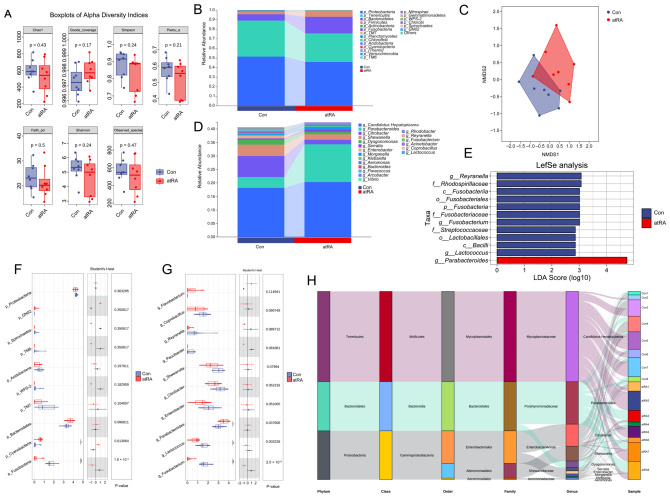
Effect of dietary atRA on the gut microbial community structure of female crabs (*n* = 8). (**A**) Alpha diversity index. (**B**,**D**) Bar graph analysis of the relative abundance of gut microbiota at the phylum and genus levels, respectively. (**C**) NMDS analysis based on beta diversity index. (**E**) Lefse analysis, with a default threshold of LDA > 2. (**F**,**G**) Inter-group difference analysis based on Student’s *t*-test at the phylum and genus levels, respectively, with “*, **, ***” indicating significant differences between two groups (*p* < 0.05 or *p* < 0.01 or *p* < 0.001). (**H**) Species composition of the mulberry plot.

As presented in Figure 5, α-diversity indices (Chao1, Simpson, and Shannon) showed no significant differences in atRA groups (Figure 5A), while β-diversity indices indicated minimal overlap between the two groups (Figure 5C). The dominant bacterial phyla were Proteobacteria, Tenericutes, and Bacteroidetes, and the dominant genera were *Candidatus Hepatoplasma*, *Parabacteroides*, and *Citrobacter* (Figure 5B,D,H). Linear discriminant analysis effect size (Lefse) results demonstrated that *Parabacteroides* was the sole biomarker microorganism specific to the atRA group (Figure 5E, LDA > 4). Further analysis using Student’s *t*-test indicated that at the phylum level, the relative abundance of Fusobacteria was extremely significantly increased (*p* < 0.001), whereas that of Cyanobacteria was significantly decreased (Figure 5F, *p* < 0.05). At the genus level, the relative abundance of *Parabacteroides* was significantly increased (*p* < 0.05), while the relative abundances of *Lactococcus* and *Fusobacterium* were extremely significantly reduced (Figure 5G, *p* < 0.01 or *p* < 0.001).

### 2.5. Effects of Dietary atRA on Fatty Acids and Amino Acids Compositions in the Hepatopancreas

**Figure 6:**
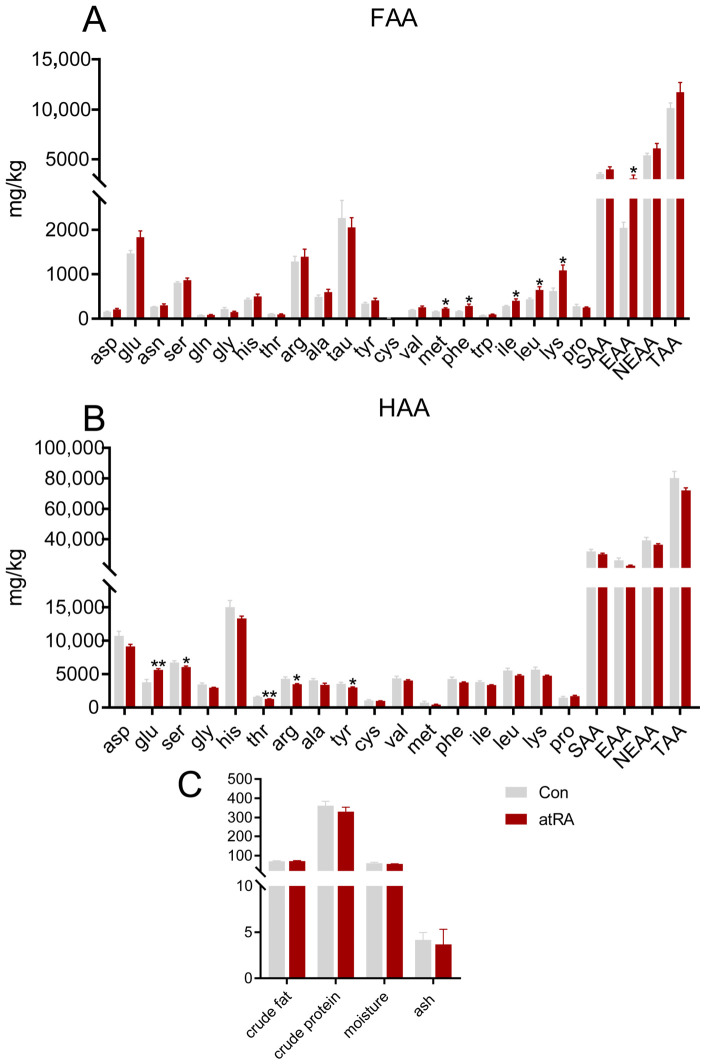
Effects of dietary atRA on the contents of free amino acids (FAAs, (**A**)), hydrolyzed amino acids (HAAs, (**B**)), and four basic nutritional components (**C**) in the hepatopancreas of female crabs (*n* = 5). Asterisks indicate significant differences between the two groups based on Student’s *t*-test (* *p* < 0.05, ** *p* < 0.01).

To further evaluate the effects of dietary atRA intervention on nutritional quality, the hepatopancreas was selected as the target tissue in the present study. As shown in Table 1, the contents of C8:0, C15:0, C17:0, C18:0, C18:1n9t, C20:1, C20:2, C20:3n6, and C20:3n3 in the atRA group were significantly decreased, whereas the contents of C16:1, C20:0, and total monounsaturated fatty acids (MUFAs) were significantly increased (Figure 6C, *p* < 0.05). Furthermore, correlation analysis also revealed that the differential fatty acids, particularly C15:0, C16:1, C18:1n9t, and C20:0, showed a significant positive correlation (Figure S2B, *p* < 0.05) with the parameters related to ovarian development (Vg, *vg*, *rxr*).

At the FAA level, atRA supplementation significantly increased the contents of methionine (Met), phenylalanine (Phe), isoleucine (Ile), leucine (Leu), lysine (Lys), and total essential amino acids (EAAs) (Figure 6A, *p* < 0.05). At the HAA level, the content of glutamic acid (Glu) was significantly elevated, whereas the contents of serine (Ser), threonine (Thr), arginine (Arg), and tyrosine (Tyr) were significantly reduced (Figure 6B, *p* < 0.05). No significant differences were observed in the four basic nutritional components: crude fat, crude protein, moisture, and ash (Figure 6C, *p* > 0.05). The correlation analysis revealed that the aforementioned differentially expressed amino acids were significantly positively correlated with the key gene *vg* involved in ovarian development (Figure S2A, *p* < 0.05).

### 2.6. The Effects of In Vivo Injection of atRA on the Related Hormones of Female Crab Ovary Development and the Analysis of Molecular Docking and TEM

**Figure 7:**
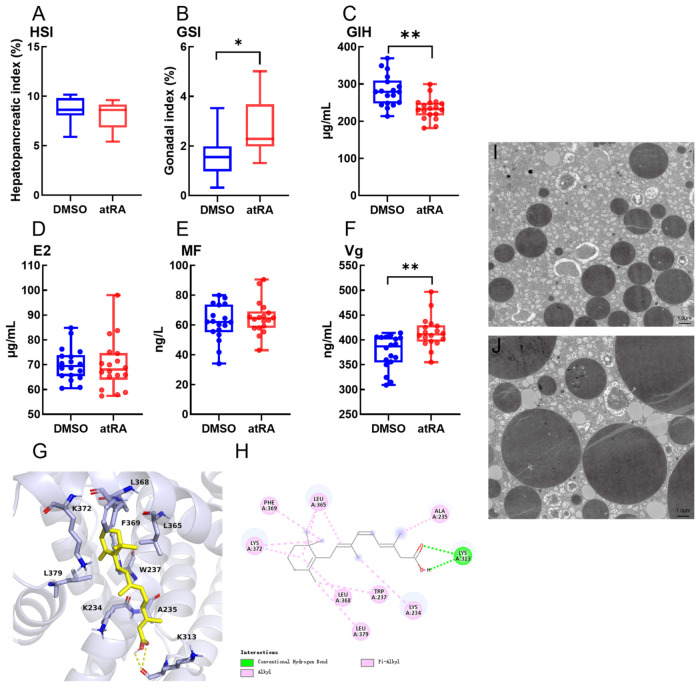
Effects of in vivo atRA injection on ovarian development-related parameters in female crabs and molecular docking analysis (*n* = 15). (**A**) HSI. (**B**) GSI. (**C**–**F**) Serum levels of GIH, estradiol (E2), methyl farnesoate (MF), and Vg, respectively. (**G**) Close-up view of the molecular docking binding site. Bright-yellow sticks represent atRA, yellow dashed lines indicate hydrogen bonds; blue and red atoms on the ligand are nitrogen and oxygen, respectively. (**H**) Two-dimensional schematic diagram of ligand–protein interactions. Asterisks indicate significant differences between the two groups based on Student’s *t*-test (* *p* < 0.05, ** *p* < 0.01). (**I**,**J**) Transmission electron microscopy (TEM) analysis of characteristic results in female crab oocytes following injection with DMSO and atRA, respectively.

In vivo injection experiments revealed that, compared with the DMSO vehicle control group, atRA treatment significantly decreased serum GIH concentration and markedly increased GSI and serum Vg level (Figure 7B,C,F, *p* < 0.05 or *p* < 0.01), although no significant differences were observed in E2, MF, and HSI (*p* > 0.05). Molecular docking results showed that the binding free energy between atRA and the ligand-binding domain of the RXR protein was −6.367 kcal/mol, indicating stable spontaneous binding between the two molecules. The terminal carboxyl group of atRA formed a specific conventional hydrogen bond with the key residue Lys313. Meanwhile, the hydrophobic scaffold of the ligand engaged in extensive alkyl and π-alkyl interactions with surrounding hydrophobic residues, including Leu234, Trp237, Ala235, Leu365, Leu368, Phe369, Lys372, and Leu379, collectively maintaining the stable conformation of the complex (Figure 7G,H). TEM analysis revealed that after atRA injection, the yolk granule size within oocytes was significantly increased, and the number of lipid droplets was elevated (Figure 7I,J).

### 2.7. The Effects of In Vivo Injection of atRA on the Development of the Ovaries, Lipid Synthesis, Catabolic Metabolism, and Transport-Related Gene Expression in Female Crabs

**Figure 8:**
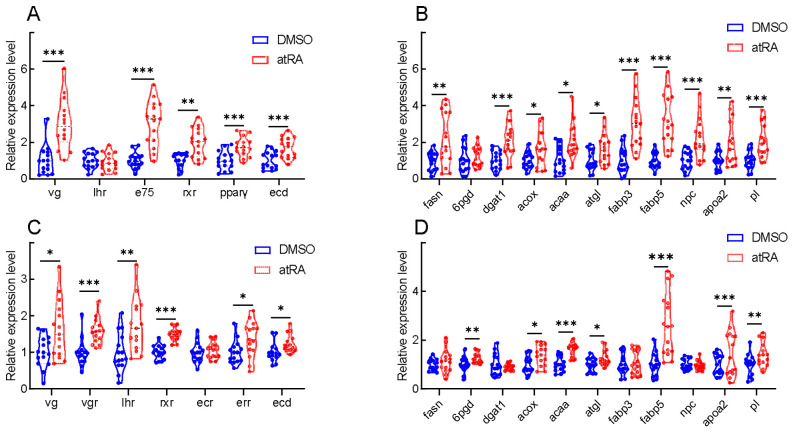
Effects of in vivo atRA injection on the expression of genes related to ovarian development, lipid anabolism, catabolism, and transport in female crabs (*n* = 15). (**A**) Core genes associated with ovarian development in the hepatopancreas, including vitellogenin (*vg*), luteinizing hormone receptor (*lhr*), ecdysone-induced protein 75 (*e75*), retinoid X receptor (*rxr*), peroxisome proliferator-activated receptor gamma (*pparγ*), and ecdysone (*ecd*). (**B**) Genes involved in lipogenesis (fatty acid synthase, *fasn*; phosphogluconate dehydrogenase, *6pgd*; diacylglycerol O-acyltransferase 1, *dgat1*), fatty acid oxidation (acyl-CoA oxidase, *acox*; acetyl-CoA acyltransferase, *acaa*), lipolysis (adipose triglyceride lipase, *atgl*), lipid transport (fatty acid-binding protein 3, *fabp3*; *fabp5*; NPC intracellular cholesterol transporter, *npc*; apolipoprotein a-II, *apoa2*), and pancreatic lipase (*pl*) in the hepatopancreas. (**C**) Core genes related to ovarian development in ovarian tissues. (**D**) Genes involved in lipogenesis, fatty acid oxidation, and lipid transport in ovarian tissues. *, **, and *** indicate significant differences at *p* < 0.05, *p* < 0.01, and *p* < 0.001, respectively, based on the Student’s *t*-test.

As shown in Figure 8A, compared with the DMSO group, atRA injection significantly upregulated the expression levels of *vg*, *e75*, *rxr*, *pparγ*, and *ecd* in the hepatopancreas (Figure 8A, *p* < 0.01 or *p* < 0.001). Similarly, the transcription of *vg*, *vgr*, *lhr*, *err*, and *ecd* in ovarian tissues was also significantly or extremely significantly increased after atRA treatment (Figure 8C, *p* < 0.05, *p* < 0.01, or *p* < 0.001). In terms of lipid metabolism, the genes involved in lipogenesis (*fasn*, *6pgd*, *dgat1*), fatty acid oxidation (*acox*, *acaa*), lipolysis (*atgl*), lipid transport (*fabp3*, *fabp5*, *npc*, *apoa2*), and pancreatic lipase (*pl*) were markedly upregulated in both the hepatopancreas and ovary following atRA injection (Figure 8D, *p* < 0.05, *p* < 0.01, or *p* < 0.001).

## 3. Discussion

### 3.1. Dietary or Injection atRA Promotes Ovarian Development in E. sinensis

The GSI is a crucial indicator for evaluating ovarian maturity. In the present study, we found that GSI increased progressively with the elevation of dietary atRA dosage, indicating that atRA has a potential role in promoting ovarian development. Notably, the dosage of 2400 mg/kg acted as a critical threshold, which significantly increased GSI. At this specific dosage, atRA supplementation led to a significant increase in oocyte diameter and enhanced lipid droplet deposition. This finding is consistent with previous reports that dietary vitamin A at a certain dosage range (8000–20,000 IU/kg) can significantly elevate GSI, increase oocyte diameter, and improve lipid droplet density [18,19]. It has been reported that GIH in aquatic crustaceans is primarily synthesized and secreted in eyestalk tissues and then acts on the ovary or seminal vesicle via hemolymph circulation to suppress the reproductive process [20]. In the present study, the expression level of the *vg* gene in the hepatopancreas was significantly higher than that in the ovarian tissue. Based on this finding, we infer that the exogenous synthesis of yolk granules in the hepatopancreas may play a dominant role. This is because during the exogenous vitellogenesis stage, Vg is mainly synthesized in the hepatopancreas and subsequently transported to the ovary through hemolymph circulation. The significant decrease in GIH and marked increase in Vg levels in the hemolymph indicated that dietary atRA or atRA injection could relieve the GIH-mediated suppression of ovarian development and accelerate yolk granule synthesis. This conclusion is further supported by the significantly upregulated *vg* gene expression in both the hepatopancreas and ovarian tissues. As a calcium-binding protein in crustaceans, Vg is tightly associated with hemolymph Ca^2+^ levels, which play a crucial role in regulating the synthesis and uptake of Vg [21]. In addition, in vitro studies have demonstrated that Ca^2+^ supplementation can increase the contents of vitellogenin/vitellin (Vg/Vn) in the hepatopancreas and ovary of *E. sinensis* [22]. In the present study, the significantly elevated Ca^2+^ concentration in the hemolymph indicated that dietary atRA could enhance Ca^2+^ absorption. This increase in Ca^2+^ may further act as a carrier to bind Vg, thereby facilitating the recognition and uptake of Vg by oocytes, or it may play a stimulatory role in the formation of endocytic vesicles [23].

RXR is a nuclear receptor that binds to the response elements of PPAR, ECR, RAR, ER, and other receptors to regulate lipid metabolism, *vg* expression, ovarian development, and embryonic differentiation. Molecular docking results revealed that atRA could stably bind to RXR, and the key residue Lys313 formed a stable terminal hydrogen bond. These findings are consistent with the report of Tsuji et al. (2015) [24], who also confirmed a high binding affinity between atRA and RXR. After the atRA injection, the relative expression levels of *rxr* and key ovarian development-related genes (including *vg*, *vgr*, *lhr*, and *ecd*) were extremely significantly upregulated in the hepatopancreas and ovary in the current study. Collectively, these results indicate that atRA can bind to RXR to elevate the transcription of *rxr* and downstream ovarian development-related genes, thereby participating in the regulation of ovarian development and highlighting the vital role of the atRA–RXR signaling axis in reproductive regulation.

### 3.2. Dietary or Injected atRA Regulates Energy Metabolism and Lipid Synthesis in the Hepatopancreas and Ovarian Tissue

AMPK acts as a cellular energy metabolic sensor. The significant upregulation of this pathway indicated that dietary atRA enhanced energy metabolism in the hepatopancreas. Studies have shown that atRA can indirectly enhance adiponectin receptor (AdipoR) activation by regulating adiponectin expression [25,26]. Activated AdipoR promotes an increase in intracellular Ca^2+^ concentration; Ca^2+^ then binds to calmodulin (CaM) to form a complex, which in turn activates Ca^2+^/calmodulin-dependent protein kinase kinase β (CaMKKβ). Activated CaMKKβ can specifically phosphorylate downstream AMPK [27]. In contrast to the classical AMPK activation mode that inhibits lipid synthesis-related genes, the genes *fas*, *acc1*, and *scd1* in the present study were significantly upregulated synchronously with AMPK. This phenomenon might be attributed to the critical stage of exogenous vitellogenesis, which requires substantial Vg synthesis in the hepatopancreas: the upregulation of *fas*/*acc1*/*scd1* drives de novo fatty acid synthesis, which preferentially supplies yolk lipid accumulation in the ovary. In the injection experiment, genes related to lipid synthesis, fatty acid oxidation, and lipid transport were also significantly upregulated in both the hepatopancreas and ovary. These results indicate that atRA supplementation can promote ovarian development by regulating lipid metabolism.

Although aquatic invertebrates lack a thyroid system, studies have demonstrated the presence of components of this system in mollusks, echinoderms, tunicates, cephalochordates, and crustaceans [28,29]. Exogenous thyroid hormone supplementation can accelerate ovarian maturation in Scylla serrata [30]. In the present study, dietary atRA upregulated the thyroid hormone synthesis and signaling pathways in both tissues. This finding implies that atRA may interact with thyroid hormone pathways associated with ovarian maturation.

### 3.3. Dietary atRA Mediates cGMP-PKG/TGF-β/Rap1/GH Signaling to Promote Oocyte Development and Maturation

In the ovary, we found that growth-related signaling pathways were significantly enriched and upregulated. Members of the TGF-β superfamily can regulate the proliferation and differentiation of spermatogonia cells in *E. sinensis*, as well as affect oocyte maturation in *S. paramamosain* and zebrafish [31,32]. The Rap1 signaling pathway and GH can regulate cell adhesion, polarity, and angiogenesis, facilitate the transport of nutrients to oocytes, maintain oocyte polarity, and thereby contribute to the initiation of embryonic development [33,34,35]. Notably, we found that the cGMP-PKG pathway was significantly enriched and upregulated at both the transcriptomic and metabolomic levels. This consistent cross-omics signature strongly suggests that it plays a central regulatory role in oocyte development. Previous studies have confirmed that the cGMP-PKG pathway serves as a key signaling axis governing the initiation of germ cell meiosis [36,37].

Furthermore, several core hub genes were identified via PPI analysis, including *smad4* (a key transcription factor in the TGF-β signaling pathway [38]), *vg*, and *acly* (a critical rate-limiting enzyme in the lipid synthesis pathway [39]). These genes were significantly upregulated in the hepatopancreas, indicating that dietary atRA can synergistically activate the regulatory network encompassing “signal transduction-nutrient synthesis-lipid metabolism”. This activation further drives the hepatopancreas to prioritize the synthesis of reproduction-related key substances (e.g., Vg and lipids), which are then directionally transported to the ovary, thereby providing essential support for exogenous vitellogenesis and ovarian maturation. In ovarian tissues, a distinct set of core hub genes, different from those in the hepatopancreas, was also identified. For instance, *slc35a2* mediates substrate transport for glycoprotein and glycolipid glycosylation [40]. *Prkag2* regulates glycolysis and lipid metabolism. *Mcm9* participates in DNA replication and damage repair [41]. *Tcp11l* maintains protein homeostasis and germ cell activity [42]. *Manf* relieves endoplasmic reticulum stress and suppresses inflammation [43]. *Sugt1* modulates protein folding and immune responses [44]. These aforementioned hub genes were significantly upregulated in ovarian tissues, indicating that dietary atRA can target and activate core hub genes associated with material metabolism, cell proliferation, protein homeostasis, and stress resistance in ovarian tissues, thereby forming a multi-pathway synergistic regulatory network that ultimately promotes oocyte maturation and ovarian development.

### 3.4. Synergistic Effects of Lipid and Nucleic Acid Metabolism Regulated by atRA on Ovarian Development

Using untargeted metabolomics, we found that pyrimidine metabolism was significantly enriched in both the hepatopancreas and ovaries, with key metabolites including thymidine, cytidine, 3′-CMP, and 5′-CMP being significantly upregulated. This indicated that dietary atRA accelerated the synthesis and turnover of pyrimidine nucleotides, thereby providing critical nucleic acid synthesis precursors for ovarian development. As is known, 5′-CMP is a direct precursor for RNA synthesis, providing a material basis for gene expression during oocyte proliferation. 3′-CMP is an intermediate product of RNA degradation, and its accumulation reflects the RNA turnover rate in cells [45]. This phenomenon indicates the dynamic balance of gene transcription in ovarian tissues and ensures the precise regulation of gene expression during oocyte maturation. Furthermore, DHEA exhibits prohormone properties; it serves as a major endogenous precursor in vivo and also acts as a critical metabolic intermediate in the process of steroid synthesis within ovarian follicles. DHEA supplementation can be used to improve ovarian reserve function. Studies have shown that DHEA not only acts as an important intermediate for the synthesis of androgens and estrogens but also functions as an “oocyte factor” that triggers calcium oscillations via the action of an endogenous agonist, thereby activating oocytes [46]. The significant elevation of DHEA levels in both the hepatopancreas and ovarian tissues suggests that dietary atRA can promote the synthesis and accumulation of DHEA. Previous studies have indicated that DHEA can enhance the atRA-induced differentiation process of promyelocytes [47]. Collectively, these findings provide valuable clues for exploring the synergistic regulatory mechanism of atRA and DHEA in reproductive functions. Etiocholanolone, a metabolic product of DHEA, exhibited an opposite expression pattern in the hepatopancreas (upregulated) and ovary (downregulated). Based on this observation, we infer that DHEA is preferentially converted into bioactive hormones (e.g., ecdysone, estrogens) in ovarian tissues. In contrast, as a primary organ responsible for metabolism and detoxification, the hepatopancreas tends to mediate the inactivation and excretion of steroids, which is supported by the significantly downregulated steroid biosynthesis pathway revealed by transcriptomic analysis. 19-OHT is an intermediate metabolite in androgen and estrogen metabolism, and it has been demonstrated to promote estradiol production when incubated with human ovarian microsomes [48]. Riboflavin-binding protein synthesized in the liver is secreted into the bloodstream, where it binds to riboflavin to form a complex. This complex is subsequently deposited as a yolk component, providing material support for oocyte development. Relevant studies in chickens have demonstrated that riboflavin is primarily derived from the diet; endogenous reserves of riboflavin are only mobilized for utilization when dietary riboflavin intake is restricted, and the riboflavin reserve in the liver exceeds 50% of the normal level [49]. The significantly elevated levels of 19-OHT and riboflavin in the hepatopancreas observed in the present study indicate that dietary atRA can activate the steroid hormone metabolic pathway and riboflavin transport and reserve mechanism in the hepatopancreas. In turn, this provides critical material and signal regulation for ovarian development and oocyte maturation through the hepatopancreas-ovary axis. ARA is a critical pro-inflammatory fatty acid substrate, and PGA2 is a metabolite derived from ARA via the cyclooxygenase pathway. The significant reduction in the ovary observed in the present study potentially indicates that dietary atRA can create an ovarian local microenvironment with “reduced pro-inflammatory lipid signaling”. This consequently induces lipid metabolism reprogramming, thereby “freeing up” more energy and carbon skeletons from the dissipation of inflammatory signaling to be preferentially allocated to support the biosynthetic processes and energy demands associated with ovarian development.

### 3.5. Regulatory Effects of atRA on Nutritional Quality and Intestinal Flora Structure

*Fusobacterium*, a potentially pathogenic bacterium [50,51], has been demonstrated to have a core metabolite, SCFAs butyrate, that can induce follicular development impairment and inhibit ovarian development [52]. In the present study, the significant reduction in the abundance of *Fusobacterium* indicated that dietary atRA can decrease the risk of disease in the host. Furthermore, Lefse analysis identified *Parabacteroides* as the sole biomarker taxon in the atRA group. This genus is recognized as a beneficial intestinal bacterium and can exert beneficial effects on host health through multiple mechanisms, including immune regulation, anti-inflammation, etc. [53,54]. For instance, relevant studies have reported that *Parabacteroides* can alleviate acute pancreatitis by producing acetate to reduce neutrophilic inflammatory infiltration [55]. *Parabacteroides* attenuates high-fat diet-induced atherosclerosis in mice by regulating lipid metabolism, inflammatory responses, and bile acid metabolism [56]. Overall, the significant increase in *Parabacteroides* abundance in the present study indicates twofold implications: on the one hand, this atRA-specific biomarker taxon may serve as a key microbial mediator of the physiological effects, particularly those associated with ovarian development; on the other hand, it suggests that dietary supplementation with a high dose of atRA does not exert adverse effects on the intestinal health of the host.

Further analysis of the conventional nutritional components in the hepatopancreas revealed that the levels of several saturated fatty acids (SFAs), namely C15:0, C17:0, and C18:0, decreased significantly following atRA treatment. Among these, C15:0 and C17:0 have been demonstrated to be positively correlated with the occurrence and development of various diseases [57]. C18:0 (stearic acid) is difficult to metabolize in the liver and may disturb the balance of hepatic nutritional metabolism [58,59]. In the present study, the significant reduction in C15:0, C17:0, and C18:0 indicates that dietary atRA can effectively decrease the accumulation of pathogenic and metabolism-burdened SFAs, thereby alleviating the functional pressure on the hepatopancreas as a lipid-metabolizing organ during the fattening period. C16:1 (palmitoleic acid) can act as a regulator of adipogenesis, desaturation, and β-oxidation in adipocytes and is also recognized as beneficial to human health [60]. C18:1n9t, similar to SFAs, is regarded as detrimental to human health and significantly correlated with an increased mortality rate induced by cardiovascular-related diseases [61,62]. The significant reduction in C18:1n9t levels, coupled with the marked increase in C16:1 and total MUFAs observed in the present study, indicates that dietary supplementation with a high dose of atRA can optimize the lipid composition and structure. Rather than disrupting the nutritional components of the hepatopancreas, this regulation exerts a beneficial effect.

The content and proportion of these EAAs, such as Met, Phe, Ile, Leu, and Lys, are recognized as the core indicators for evaluating the nutritional value of food proteins. After dietary atRA, the levels of these EAAs in the hepatopancreas increased significantly in the present study. In particular, the elevated content of Lys can effectively improve the overall utilization efficiency of dietary proteins. Leu and Ile regulate energy homeostasis, nutrient metabolism, intestinal health, and immune function via the PI3K/AKT/mTOR signaling pathway [63]. Met participates in intracellular methylation reactions and plays an important role in maintaining DNA stability and improving lipid metabolism [64,65]. Phe can be converted into tyrosine in the human body, which is further metabolized to synthesize neurotransmitters and bioactive substances. This conversion process plays a positive role in maintaining neurological function and improving skin health [66]. Overall, at the amino acid level, dietary atRA not only does not disrupt the amino acid composition but also significantly increases the contents of various key EAAs in the hepatopancreas, thereby comprehensively optimizing its nutritional quality for human consumption and providing a higher-quality amino acid source for the human body.

## 4. Materials and Methods

### 4.1. The Sources of Experimental Materials, Experimental Design, and Sample Collection for the Feeding Experiment

Female crabs that had completed their reproductive molting were obtained from the Yangzhong Base of the Freshwater Fisheries Research Center, Chinese Academy of Fishery Sciences. A total of 300 crabs with vigorous vitality and intact limbs were selected for the experiment. They were first acclimated to the experimental conditions briefly in an indoor breeding barrel (diameter 2 m, height 0.8 m). In the current study, a basal diet (0 atRA) was formulated using fish meal and soybean meal as the primary protein sources and fish oil as the lipid source. Based on this formulation, varying doses of atRA (Shanghai Macklin Biochemical Technology Co., Ltd., Shanghai, China) were supplemented to the basal diet to achieve atRA concentrations of 150–2400 mg/kg diet (designated as 150 atRA, 300 atRA, 600 atRA, 1200 atRA, and 2400 atRA). All feed ingredients were ground to pass through a 60-mesh sieve, thoroughly mixed according to the feed formulation, and combined with distilled water. The mixture was then processed using a pelletizer to produce pellets with a diameter of 4.5 mm and a length of approximately 8 mm. The resulting pellets were air-dried at room temperature and subsequently stored at −20 °C. The formulation of the experimental diets is presented in Table S1, and the proximate composition analysis of the diets is provided in Table S2. The atRA concentrations used in this study were determined based on previously published research [9,67].

Following acclimation, 270 female crabs of similar size (125.57 ± 22.56 g) were randomly allocated to the six aforementioned groups. Each group included three replicates, with 15 crabs per replicate. Subsequently, daily feeding was conducted at 17:00 each day, with a feeding rate of 3–5% of body weight. Two hours after feeding, residual feed and excreta were removed, and one-third of the water was changed. During this period, the temperature was maintained at 25–28 °C, and the dissolved oxygen (DO) content was ≥5 mg/L. Ammonia nitrogen (<0.2 mg/L), nitrite nitrogen (<0.005 mg/L), and hydrogen sulfide (<0.01 mg/L) were regularly monitored and controlled within safe limits.

The experiment was conducted over seven weeks, and feeding was halted one day before sampling. Five female crabs were randomly selected from each replicate, leading to a total of 15 crabs per group for sample collection. Subsequently, hemolymph was extracted from the basal membrane of the third leg of the crab using a disposable medical syringe (1 mL). Sterile scissors and forceps were then used to remove the hepatopancreas, ovary, and intestinal tissue. The hepatosomatic index (HSI) and gonadosomatic index (GSI) were calculated as (hepatopancreas or ovary weight/body weight) × 100%, respectively. The samples were promptly frozen at −80 °C for subsequent analysis.

### 4.2. Intravenous Injection Experimental Design and Sample Collection

One hundred female crabs with completed reproductive molting (body weight: 131.96 ± 23.93 g) were first acclimated in an indoor recirculating aquaculture system for seven days to adapt to the experimental conditions. After acclimation, 90 crabs were randomly divided into six culture systems. Female crabs in three systems were injected with 10% DMSO (vehicle solvent), prepared by mixing one part DMSO with nine parts physiological saline. For atRA injection, a stock solution of 30 mg/mL was first prepared in 100% DMSO, which was subsequently diluted with 10% DMSO in physiological saline to a working concentration of 0.15 mg/mL. The injection volume was 2 μL/g live mass, and the injection site was the membrane at the base of the third pereiopod. Overall, the atRA injection was administered strictly according to body weight at a dose of 0.3 μg/g (i.e., 300 μg/kg), which was referenced from the study by Venkaiah et al. (2019) [68]. The injection was performed once every seven days for a total of five times. Sample collection began on the 5th day after the last injection. During the experiment, the feeding regime and water quality management were consistent with those of the feeding trial.

After the 35-day injection experiment, seven crabs were randomly selected from each biological replicate, with 21 individuals collected per group for subsequent sampling. Hemolymph, hepatopancreas, and ovarian tissues were aseptically dissected. The hepatopancreas and ovarian tissues were weighed to calculate the HSI and GSI. Partial ovarian tissues were fixed in 2.5% glutaraldehyde for subsequent transmission electron microscopy (TEM) observation. The remaining tissue samples were immediately frozen in liquid nitrogen and stored at −80 °C until use.

### 4.3. Enzyme Activities and ELISA Analysis

The hemolymph was centrifuged at 6000 rpm for 10 min at 4 °C to obtain the supernatant for subsequent analysis. The kits for Ca^2+^ (C004-2-1), K^2+^ (C001-2-1), AKP (A059-2-2), TG (A110-1-1), and T-CHO (A111-1-1) were purchased from Jiancheng Bioengineering Institute (Nanjing, China). The sandwich ELISA kits for gonad-inhibiting hormone (GIH), estradiol (E2), methyl farnesoate (MF), and Vg were obtained from Jiangsu Meimian Industrial Co., Ltd. (Yancheng, China). The competitive assay atRA test kit can be purchased from Beijing Huabodeyi Biology Science and Technology Co., Ltd. (Beijing, China). All the above tests were conducted strictly following the manufacturer’s instructions.

### 4.4. Transcriptomic Analysis of the Hepatopancreas and Ovary

Regarding the feeding experiment, based on the analysis of GSI, enzyme activities, and ELISA data, it was found that the highest dose of the atRA diet significantly promoted ovarian development and accelerated Ca^2+^ accumulation. Therefore, the 2400 mg/kg group and the control group were selected for multi-omics sequencing (including transcriptomics, non-targeted metabolomics, and 16s rDNA sequencing) to further explore key signaling pathways, differential genes, metabolites, and microorganisms. For transcriptomic analysis, three individual samples were randomly pooled to form one biological replicate. Consequently, five biological replicates were obtained for each tissue type in both the control group and the 2400 mg/kg group (*n* = 5); this also applies to the following untargeted metabolomics analysis. A total of 20 samples were subjected to total RNA extraction following the Trizol method. Detailed steps such as subsequent library construction, high-throughput sequencing on the NovaSeq 6000 platform (Illumina, Shanghai Personal Biotechnology Co., Ltd., Shanghai, China) with paired-end reads with a length of 150 bp (PE150), sequencing data quality control, and sequence alignment analysis were performed as described in our previous study [18]. Gene expression levels were quantified as fragments per kilobase of transcript per million fragments mapped (FPKM). Differentially expressed genes (DEGs) were defined as those with a false discovery rate (FDR) < 0.05 and an absolute value of fold change (|FC|) ≥ 2. Subsequently, functional enrichment analysis, including Gene Ontology (GO) and Kyoto Encyclopedia of Genes and Genomes (KEGG), is performed on the DEGs based on PICRUSt2 software (version 2.2.2-b).

### 4.5. Total RNA Extraction, cDNA Synthesis, and RT-PCR

Total RNA (*n* = 15) was extracted from all samples using the Trizol reagent (Takara, Takara Bio Inc., Shiga, Japan) method. Qualified RNA was reverse transcribed into cDNA with the PrimeScript™ FAST RT Kit (Takara, Takara Bio Inc., Shiga, Japan). Subsequently, quantitative real-time PCR was performed using TB Green Fast mix (Takara, Takara, Takara Bio Inc., Shiga, Japan) on the CFX96 real-time detection system (Bio-Rad Laboratories, Inc., Hercules, CA, USA). The *β-actin* gene was selected as the internal reference gene. The relative expression levels of target genes were calculated via the 2^−ΔΔCt^ method. All primer sequences used in this study are provided in the Supplementary Materials Table S3.

### 4.6. Non-Targeted Metabolomics of the Hepatopancreas and Ovary

Non-targeted metabolomic analysis was also conducted based on the aforementioned 20 samples (*n* = 5). Firstly, approximately 100 mg of frozen tissue samples were weighed and added to 1 mL of precooled extraction solution (methanol-acetonitrile-water, volume ratio 2:2:1). After low-temperature mechanical homogenization using a homogenizer and ultrasonic extraction in an ice-water bath, the samples were incubated at −20 °C for 60 min to precipitate proteins and then centrifuged at 13,000× *g* for 15 min at 4 °C. The supernatant was collected, concentrated under vacuum, and vacuum dried, and the lyophilized powder was stored at −80 °C for future use. Using an Agilent 1290 Infinity UHPLC system (Agilent Technologies, Inc., Santa Clara, CA, USA), coupled with a Waters ACQUITY UPLC BEH Amide chromatographic column (column temperature 25 °C) (Waters Corp., Milford, MA, USA), an aqueous solution containing 25 mM ammonium carbonate and ammonia water (phase A)–acetonitrile (phase B) was used as the mobile phase. Data were collected using a Triple TOF 6600 mass spectrometer (SCIEX, Framingham, MA, USA) in positive and negative ion ESI modes. The data were normalized based on the total peak area, and metabolite features with missing values > 50% within the group were eliminated. SIMCA-P 16.1 software was used for multivariate statistical analysis of PCA and OPLS-DA. Differentially expressed metabolites (DEMs) with Variable Importance in Projection (VIP) > 1.0, *p* < 0.05, and |FC| ≥ 2 were screened. KEGG pathway annotation and enrichment analysis were performed on the DEMs, and their expression patterns were displayed through hierarchical clustering analysis.

### 4.7. 16s rDNA Sequencing Analysis

For the 16s rDNA analysis of intestinal contents, 8 samples were randomly selected from each group, totaling 16 samples for DNA extraction (*n* = 8). PCR amplification, library construction, and high-throughput sequencing were performed according to our previously published study [69]. In brief, the V3-V4 region of the 16S rDNA gene was amplified using specific primers 338F (5′ barcode ACTCCTACGGGAGGCAGCA-3′) and 806R (5′ GGACTACHVGGGTWTCTAAT-3′). The library was constructed using the TruSeq Nano DNA LT Kit (Illumina); quality control was performed using a high-sensitivity DNA kit (Agilent Technologies), and quantification was done using the Quant-iT Pico Green Kit (Thermo Fisher Scientific, Waltham, MA, USA). Subsequently, the qualified library underwent 2 × 250 bp dual-end sequencing on the Illumina NovaSeq 6000 platform. The demultiplexed sequencing data were denoised using the R 4.3.3 package DADA2. The ASV feature sequences and abundance table were then merged, and singletons were removed. Species annotation of ASVs was performed based on the Greengenes database. Bar plots showing the taxonomic composition at the phylum and genus levels for each sample were generated using R. PERMANOVA/Adonis analysis was used to test for significant differences in microbial community structure among groups. Alpha diversity indices were calculated using QIIME2, and the Kruskal–Wallis test was applied to determine significant differences between groups. Non-metric Multidimensional Scaling (NMDS) analysis based on Bray–Curtis distance was performed using the vegan package. Biomarkers between groups were identified using the Lefse install package based R 4.3.3, and a histogram illustrating their distribution based on LDA values (>2) was subsequently plotted.

### 4.8. Hematoxylin-Eosin (H&E) Staining and TEM Analysis of Ovarian Tissue

Ovarian tissue samples (*n* = 3) from both the control group and the highest atRA dosage group were fixed first, then subjected to a standard histology protocol involving gradient ethanol dehydration, paraffin embedding, and sectioning to a uniform thickness of 6 μm with a rotary microtome for subsequent morphological observation. Subsequently, based on xylene dewaxing and ethanol rehydration, H&E staining was performed. After another gradient ethanol dehydration, xylene was used to make the sections transparent. Finally, neutral resin was used for mounting, and observation and statistics were conducted under an optical microscope.

For TEM analysis, ovarian tissues (*n* = 3) were fixed in glutaraldehyde, followed by secondary fixation in 1% osmium tetroxide for 2 h at room temperature in the dark. After washing with PBS, the samples were dehydrated using a graded ethanol series (30% to 100%) and then treated with acetone. Subsequently, the tissues were infiltrated with acetone and 812 resin mixtures at progressively increasing resin ratios, embedded in pure 812 resin, and polymerized first at 37 °C overnight and then at 60 °C for 48 h. Ultrathin sections (60–80 nm in thickness) were double-stained with uranyl acetate (8 min, in the dark) and lead citrate (8 min, under CO_2_-free conditions) and finally examined using transmission electron microscopy.

### 4.9. Nutritional Quality Analysis

The detection of proximate composition, including moisture (GB/T 5009.3-2016) [70], ash (GB 5009.4-2016) [71], crude protein (GB/T 6432-2018) [72], and crude fat (GB 5009.6-2016) [73], in hepatopancreatic tissues (*n* = 5) from atRA and control groups was conducted. Each test sample was pooled from three replicates. The determination of free/hydrolyzed amino acids (FAAs/HAAs) and 37 fatty acids (*n* = 5) in hepatopancreatic tissues was also completed with reference to our previous study [15].

### 4.10. Molecular Docking

In the present study, a semi-flexible docking approach was employed to further investigate the binding mode between the small molecule atRA and the RXR target protein. Docking was performed using AutoDock Vina 1.2.7 software, where atRA (PubChem CID: 444795) was docked against RXR. The amino acid sequence of RXR was obtained from the National Center for Biotechnology Information (NCBI), and the protein structure was predicted using AlphaFold3 (https://alphafoldserver.com/). The protein structure was then preprocessed in PyMOL 3.0.3 to remove water molecules and non-relevant ligands, followed by hydrogen atom addition. The compound was subjected to energy minimization using ChemDraw 20.0. Subsequently, PDBQT files were generated with AutoDock Tools 1.57, and the grid box was set to cover the entire protein structure, with all other parameters kept as default. Nine conformations were generated per docking run, and the conformation with the lowest binding energy and highest cluster frequency was selected as the most probable binding mode. Finally, the docking results were visualized using PyMOL 3.0.3 to intuitively display the binding conformation and interactions between the ligand and the target protein.

### 4.11. Statistical Analysis

All data were expressed as mean ± standard error. If the data conformed to a normalized distribution (Kolmogorov–Smirnov test) and homogeneity of variance (Levene test), one-way ANOVA was used for comparisons among multiple groups. Otherwise, a nonparametric test (Kruskal–Wallis test) was employed. For comparisons between two groups, Student’s *t*-test or Mann–Whitney U test (when data do not satisfy normal distribution) was used. Bonferroni correction was adopted for post hoc pairwise comparisons, and *p* < 0.05 was considered statistically significant. Data analysis was conducted using SPSS software 26.0 and visualized using GraphPad Prism 8.0.2 software.

## 5. Conclusions

This study systematically evaluates the regulatory effects of atRA on ovarian development, hepatopancreatic metabolism, intestinal microbiota, and nutritional quality of female crabs. The results demonstrated that dietary atRA dose-dependently promoted ovarian development, with 2400 mg/kg identified as the critical effective dose. In addition, molecular docking revealed that atRA could spontaneously bind to RXR. In vivo injection experiments demonstrated that atRA activated *rxr* and, by regulating lipid metabolism processes, induced ovarian development and maturation. Meanwhile, the atRA diet remarkably optimized the amino acid and lipid composition of the hepatopancreas, reduced the accumulation of harmful fatty acids, and increased the proportion of beneficial fatty acids and various key EAAs. Dietary atRA exerted a positive regulatory effect on the intestinal microbiota structure by reducing the abundance of potential pathogenic bacteria and enriching the beneficial bacterium *Parabacteroides*. Overall, this study demonstrates that atRA supplementation benefits ovarian development, nutritional quality, and intestinal microbial community structure of female crabs during the fattening period. However, this study still has some limitations. No additional vehicle controls were included in the injection experiment. The dietary trial lacked positive controls for ovarian maturation and paired feeding controls; thus, we cannot rule out the possibility that atRA indirectly regulates ovarian development by affecting appetite.

## Figures and Tables

**Table 1:** Effects of dietary atRA on fatty acid composition in the hepatopancreas of female crabs (*n* = 5).

Fatty Acids (%)	Con	atRA	*p*-Value
C8:0	0.001 ± 0.000	0.001 ± 0.000	0.025
C10:0	0.006 ± 0.004	0.004 ± 0.001	0.298
C11:0	0.004 ± 0.001	0.004 ± 0.001	0.241
C12:0	0.081 ± 0.008	0.089 ± 0.008	0.131
C13:0	0.036 ± 0.008	0.036 ± 0.005	0.973
C14:0	1.253 ± 0.125	1.313 ± 0.107	0.439
C14:1	0.383 ± 0.061	0.414 ± 0.031	0.332
C15:0	0.603 ± 0.079	0.515 ± 0.029	0.047
C16:0	20.595 ± 1.318	20.716 ± 0.953	0.873
C16:1	5.850 ± 1.480	8.011 ± 0.842	0.022
C17:0	0.520 ± 0.045	0.382 ± 0.036	0.001
C18:0	3.474 ± 0.477	2.684 ± 0.188	0.009
C18:1n9t	0.244 ± 0.014	0.205 ± 0.020	0.007
C18:1n9c	31.909 ± 1.672	32.528 ± 0.807	0.478
C18:2n6c	20.900 ± 1.564	20.464 ± 1.709	0.685
C20:0	0.067 ± 0.008	0.091 ± 0.015	0.014
C18:3n6	2.312 ± 0.444	2.327 ± 0.200	0.947
C20:1	0.370 ± 0.039	0.288 ± 0.028	0.005
C18:3n3	1.348 ± 0.229	1.317 ± 0.140	0.801
C21:0	1.486 ± 0.284	1.329 ± 0.175	0.322
C20:2	0.138 ± 0.017	0.110 ± 0.009	0.011
C22:0	0.250 ± 0.060	0.244 ± 0.058	0.872
C20:3n6	1.605 ± 0.323	1.113 ± 0.161	0.016
C22:1n9	0.379 ± 0.055	0.398 ± 0.073	0.665
C20:3n3	0.269 ± 0.045	0.196 ± 0.029	0.015
C20:4n6	2.117 ± 0.236	1.898 ± 0.304	0.239
C23:0	0.345 ± 0.096	0.314 ± 0.058	0.547
C22:2n	0.008 ± 0.002	0.011 ± 0.002	0.055
C24:0	0.128 ± 0.019	0.132 ± 0.005	0.670
C20:5n3	0.130 ± 0.034	0.100 ± 0.023	0.144
C24:1	0.088 ± 0.031	0.078 ± 0.021	0.565
C22:6n3	3.099 ± 1.017	2.689 ± 0.414	0.428
MUFA	39.223 ± 1.269	41.922 ± 1.509	0.016
PUFA	31.926 ± 2.620	30.225 ± 2.003	0.282
SFA	28.850 ± 1.405	27.854 ± 1.091	0.246

## Data Availability

The datasets used and/or analyzed during the current study are available from the corresponding authors on reasonable request.

## Supplementary Materials

The following supporting information can be downloaded at: https://www.mdpi.com/article/10.3390/ijms27115148/s1.

## Abbreviations

The following abbreviations are used in this manuscript:

ATRA	All-trans retinoic acid
GSI	Gonadosomatic index
GIH	Gonad-inhibiting hormone
TEM	Transmission electron microscopy
ELISA	Enzyme-linked immunosorbent assay
DEGs	Differentially expressed genes
FC	Fold change
RT-PCR	Quantitative real-time PCR
DEMs	Differentially expressed metabolites
GO	Gene Ontology
KEGG	Kyoto Encyclopedia of Genes and Genomes
FDR	False discovery rate
PCA	Principal component analysis
DMSO	Dimethyl sulfoxide
FAAs	Free amino acids
HAAs	Hydrolyzed amino acids
