# Supplementary material for: Effects of All-Trans Retinoic Acid on Ovarian Development, Lipid Metabolism, Nutritional Quality, and Gut Microbiota of Female Chinese Mitten Crab During Fattening Period
Source: Int J Mol Sci. 2026 Jun 5;27(11):5148. doi: 10.3390/ijms27115148 (PMC13258675; doi:10.3390/ijms27115148)
Supplement: Supplementary file 1 [file ijms-27-05148-s001.zip › ijms-4335684-supplementary.pdf]

Table S1. Percentage of ingredients in the experimental feed formula.

Item	0atRA	150atRA	300atRA	600atRA	1200atRA	2400atRA
Fish meal	25.00	25.00	25.00	25.00	25.00	25.00
Squid meal	4.00	4.00	4.00	4.00	4.00	4.00
Soybean meal	18.00	16.00	16.00	16.00	16.00	16.00
Peanut meal	6.00	5.50	5.50	5.50	5.50	5.50
Rapeseed meal	5.00	5.50	5.50	5.50	5.50	5.50
Shrimp paste	3.00	3.00	3.00	3.00	3.00	3.00
Wheat flour	6.00	6.00	6.00	6.00	6.00	6.00
Rice bran	4.00	4.00	4.00	4.00	4.00	4.00
Pregelatinized starch	10.00	10.00	10.00	10.00	10.00	10.00
Fish oil	3.50	3.50	3.50	3.50	3.50	3.50
Glutamate	0.80	0.80	0.80	0.80	0.80	0.80
Taurine	0.25	0.25	0.25	0.25	0.25	0.25
Choline chloride	0.45	0.45	0.45	0.45	0.45	0.45
Vitamin premix[a]	0.50	0.50	0.50	0.50	0.50	0.50
Mineral premix[b]	0.70	0.70	0.70	0.70	0.70	0.70
$Ca(H_2PO_4)_2$	1.10	1.10	1.10	1.10	1.10	1.10
Ethoxyquin	0.02	0.02	0.02	0.02	0.02	0.02
Propionic acid	0.03	0.03	0.03	0.03	0.03	0.03
Yttria	0.10	0.10	0.10	0.10	0.10	0.10
atRA	0.000	0.015	0.030	0.060	0.120	0.240
Cellulose	0.300	0.285	0.275	0.240	0.180	0.060

Notes: a Vitamin premix (IU or mg/kg diet): Vitamin A 1500 IU, VD3 4600 IU, VE 350 IU, VK3 22 mg, VB1 60 mg, VB2 90 mg, VB6 85 mg, VB12 0.11 mg, biotin 1.8 mg, D-calcium Pantothenate 160 mg, folic acid 16 mg, VB3 230 mg, VC 1000 mg, inositol 570 mg.

b Minerals premix (mg/kg diet): FeSO4·H2O 150, CuSO4·5H2O 70, ZnSO4·H2O 280, MnSO4·H2O 80, MgSO4·H2O 180, KH2PO4 3200, NaH2PO4 400, Ca(IO3)2 4.2, CoCl2·6H2O 1.6, Na2SeO3 2.2, Mg 130.

Table S2. Approximate ingredients of feed in current study.

Group (atRA, mg/kg)	Crude protein (g/kg)	Crude fat (%)	Ash (g/100g)	Moisture (%)
0	501.06 ± 13.80	6.30 ± 0.23	13.47 ± 0.03	37.68 ± 0.13
150	504.91 ± 10.23	6.63 ± 0.16	14.65 ± 0.36	37.47 ± 0.03
300	488.97 ± 15.69	6.51 ± 0.09	13.33 ± 0.38	37.58 ± 0.03
600	509.78 ± 17.63	6.79 ± 0.09	15.14 ± 0.12	37.84 ± 0.02
1200	514.31 ± 11.12	6.44 ± 0.17	14.91 ± 0.02	38.22 ± 0.21
2400	503.34 ± 7.03	6.66 ± 0.27	14.63 ± 0.03	38.05 ± 0.44

Table S3. The primer sequence information for the current study

Gene	Forward	Reverse	Accession No.
vg	CCTGCAGATGGTTGAATACCAAG	TTGATCGGCAGAATGAACTCAGG	XM_050848579.1
vgr	TTCCAAAGCCTCCATCTCATGTT	AGGTGTCGTTCTTACACTTTCCA	XM_050859828.1
lhr	CTACTCCTCTGTCAACAACACCT	GGTTGGAGTCCTCTGAAGTGATT	MK312165.1
pparγ	GCATCTCTGATTCCAGGCTATGA	TTAGGTTCCCGTATCTCTGGTCT	XM_050882524.1
e75	TGAACTGTGTGTGTGTCGTGTTA	ATATGTCCCAGGTTCACGAAGAG	XM_050855452.1
rxr	CTTGACCATGGGGATGAAGAGA	CTGCTCATCTATGGGTTCCACA	XM_050872182.1
ecd	TGAGTGAGACCTGTGTTAGTGTG	GGAGATGCCACTAATACCCTACG	XM_050836155.1
ecr	CCCCATTAGTCCAGTGAGTAAAGA	TTATATCCGCTTCAGTTGGCTGT	XM_050870799.1
err	TTCACCGAACTAGCACTGAATGA	TTGCATTCTCTTGCTTGCTTCTC	XM_050859816.1
fasn	TTTCTGAAGGTGGGAATGGGAGGGGA	GGCCTGTTGTGGTGCCATGTTCAAA	KM042200.1
6pgd	CCTTCAACAGAACGACGGAGAA	CAGCAGAGGGATTAGCTTCTCAA	XM_050880455.1
dgat1	AACCGGAAGATCCCTCAGGA	GGGATCCTGATGGGTGCAAA	MF497736.1
acox	ACTTTGTTATGTTCATGCCAGCC	GAGAGTGCAACACAAACTCCTG	XM_050840879.1
acaa	GAGGGAGTGGGAGACATACTTG	TTCCTCCACTGGCATTGTTTTC	XM_050851879.1
atgl	CACCTTTGTACTGCAACACTCTG	AAGGTAATCCCTGATGGCTCTTG	XM_050835376
fabp3	AAGTACGTCCTGGAGACCTCTG	CTTGAGGGTGGAGATGGTCTTG	KJ804230.1
fabp5	CATACAAACTGGAGAGGAGCGAT	TGAAACTCCAGGTAAGGGTTGAG	XM_050840796.1
npc	TCGATGTCTCTGGAGGATGAGG	CTCCGTATGAAGCGTGGATCTG	XM_050846459.1
apoa2	AAGATGGATGATGTTCGTGATTGG	TTTTCGTACAGGTCGCTGAAAGT	XM_050869131.1
pl	TGACTACGATTCCTTTGAGCTGG	TGACCTGGTAATGGTACAAGCAG	MG456862.1
β-action	TCATCACCATCGGCAATGA	TTGTAAGTGGTCTCGTGGATG	XM_050843216.1

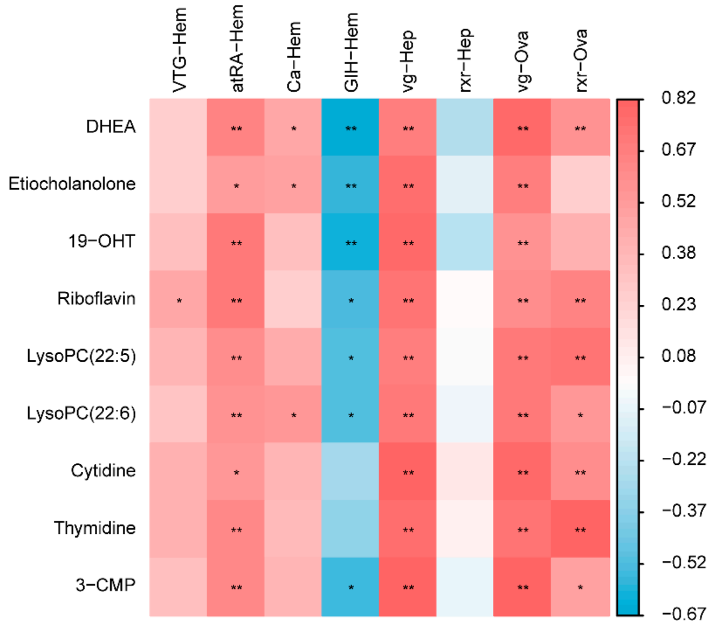

Figure S1. Pearson correlation analysis between differentially abundant metabolites in the hepatopancreas and parameters related to ovarian development. The abbreviations Hem, Hep, and Ova on the horizontal axis represent hemolymph, hepatopancreas, and ovary, respectively.

In the heatmap, red indicates positive correlation, blue indicates negative correlation, and *, ** denote $P < 0.05$ and $P < 0.01$, respectively.

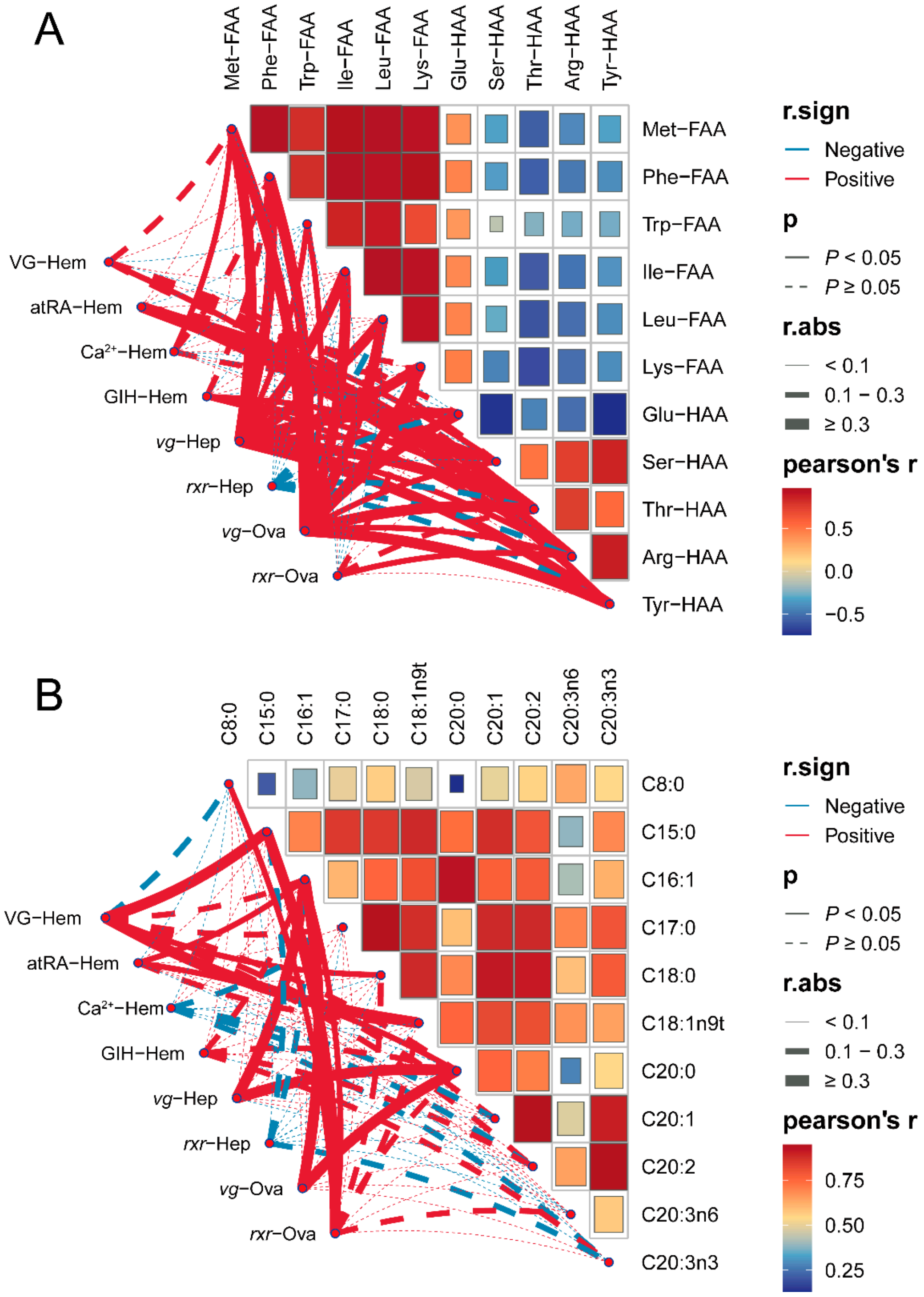

Figure S2. Pearson correlation analysis between differentially amino acids (A), fatty acids (B) in the hepatopancreas and parameters related to ovarian development. The abbreviations FAA, HAA, Hem, Hep, and Ova represent free amino acids, hydrolyzed amino acids, hemolymph, hepatopancreas, and ovary, respectively. In the heatmap, red indicates a positive correlation between fatty acids or amino acids, while blue indicates a negative correlation.
